# Needs and experiences of postgraduate nursing students in Nigeria during the COVID‐19 pandemic

**DOI:** 10.1002/nop2.70031

**Published:** 2024-09-06

**Authors:** Oluwadamilare Akingbade, Victoria O. Faremi, Chioma J. Eze, Chioma B. Eze, Esther Oluwasola, Samuel A. Olawoore, Victoria Adediran, Oluwatobi B. Kolawole, Emmanuel O. Adesuyi

**Affiliations:** ^1^ Faculty of Nursing University of Alberta Edmonton Alberta Canada; ^2^ Institute of Nursing Research Osogbo Nigeria; ^3^ School of Nursing and Midwifery Birmingham City University Birmingham UK

**Keywords:** COVID‐19 pandemic, Nigeria, postgraduate nursing students, qualitative study

## Abstract

**Aim:**

To explore the experiences and needs of postgraduate nursing students within the Nigerian context.

**Design:**

This qualitative study was conducted using a descriptive phenomenological approach.

**Method:**

Data were collected between February and April 2022 using a purposive sampling method and telephone semi‐structured interviews. Colaizzi's method of Qualitative data Analysis was utilized. Twenty‐two Nigerian postgraduate nursing students were interviewed.

**Results:**

Three themes emerged: challenges of Nigerian postgraduate students before the pandemic, the impact of the pandemic on postgraduate education, and innovations to improve postgraduate education in Nigeria. The challenges include the burden of physical lectures, lack of infrastructure, and poor mentorship of postgraduate nursing students. The impact of the pandemic on postgraduate education includes abrupt disruption of the academic program, a prolonged academic calendar, and a communication gap between students and their research supervisors. Innovations to improve postgraduate nursing education in Nigeria also include adoption and sustainability of e‐learning, upgrading post‐basic to postgraduate nursing programmes, proper structuring of postgraduate nursing education, commencement of postgraduate nursing programmes in more universities and provision of financial aid for students. Our primary finding is that funding, mentorship and infrastructure were issues peculiar to all the respondents.

**Conclusion:**

This study concludes that efforts should be made to maintain a seamless educational program by ensuring an uninterrupted flow of learning through virtual means, thereby enhancing effective teaching and learning.

**Implications:**

Graduate nursing studies is one of the suggested solutions in the WHO strategic direction for nursing and midwifery globally to achieve Universal Health Coverage . The reason is that nurses can practice with more and better skills in any work setting, thus improving the quality of health care services. Our study provides insights into the experiences of postgraduate students and how these could discourage other nurses who might have thought about furthering their studies. Efforts should be made to provide all the support that these students need, using evidence from this study and similar studies to ensure they have a good learning experience and others can be motivated to learn at the graduate level as well. This will increase the proportion of nurses and midwives honed with better skills to provide more standard quality services that will improve patient care outcomes.

## 1 INTRODUCTION

Before the pandemic, learning and studying for postgraduate students in Nigeria was mainly face‐to‐face (Ojo Joseph et al., 2021). Whereas, for more developed countries, the virtual form of learning has been used increasingly to complement the face‐to‐face pattern of education before the COVID‐19 pandemic, the same cannot be said of Nigeria (Kyari et al., 2018). Ifijeh et al. (2015) argued that few higher education institutions in Nigeria used e‐learning platforms to make lecture notes available to students and assignments before the pandemic. Similarly, Eze et al. (2018) affirmed that Nigeria's traditional face‐to‐face learning style required students to attend classes, workshops, tests, examinations, and other academic activities physically in classrooms on campus.

Coronavirus (SARS‐Cov‐2), first discovered in Wuhan, China, broke out fast around the end of 2019 and affected the whole world, especially the educational sector (Afolalu et al., 2021; Atekoja et al., 2020). Following the pandemic, Nigeria, like other countries, employed various measures to control the spread of the pandemic, including the complete closure of educational institutions (Okondu et al., 2021; Oyewumi et al., 2022). The closure of educational institutions in Nigeria has many impacts because the education system worked on substandard platforms before the pandemic. Such impacts include a lack of access to relevant study materials, a lack of proper mentorship and guidance from lecturers, and the lack of motivation and willingness to keep studying (Adejumo et al., 2023). This dismal in postgraduate education must have been slightly exaggerated since digital technology has remarkably influenced students' experiences. Adeyanju et al. (2022) argued that poor infrastructural facilities, especially Information and Communication Technology (ICT) and the internet in Nigerian higher institutions, made the transition to online teaching and learning difficult.

The first postgraduate nursing program in Nigeria began in 1988 at Obafemi Awolowo University, followed by similar programs at the University of Ibadan and the University of Nigeria, Nsukka (Onwe, 2018). These three federal universities led the way in postgraduate nursing education for many years, with several state and private institutions later joining the effort. Despite this growth, postgraduate nursing programmes in Nigeria have faced numerous challenges. Between 1988 and 2014, only 88 out of 880 postgraduate nursing students (MSc and PhD) completed their degrees ( Adeleye, et al., 2023; Onwe, 2018). Although there is limited documentation on the state of postgraduate nursing education in Nigeria, Onwe (2018) highlighted concerns from the Nigerian government and professional stakeholders, including the Nursing and Midwifery Council of Nigeria, about the low graduation rates (10%) and high attrition rates (20%) in relation to the standard programmme durations of one year for a master's degree and three years for a PhD. Contributing factors include poor funding, lack of scholarships, difficulties in balancing full‐time work with study, faculty workload and development issues and policy challenges related to program structure and implementation.

Jimoh‐Kadiri and Bupo (2011) claimed that virtual learning gradually gained recognition in higher educational institutions due to the sporadic development in internet technology. It is difficult to think that a time will come in the history of education worldwide, particularly in nursing, when virtual platforms will become the indispensable means of sustaining educational priorities. Students can now access teaching and learning content anytime and anywhere without being in class or the physical presence of a lecturer (Wallace et al., 2021). Even though technology has been instrumental in removing geographical barriers to learning posed by the COVID‐19 pandemic restrictions, its overall impact on teachers, learners and learning outcomes is still unknown (Egoigwe et al., 2020).

García‐Morales et al. (2021) rightly used the word “experimentation” to summarize the quick adoption of virtual learning platforms at the height of the COVID‐19 pandemic. This has generated genuine concern over the quality of online learning and students' academic performance because of limited available information to guide best practices for online education (Sahu, 2020). Egoigwe et al. (2020) reported similar concerns over the academic performance of postgraduate students in Nigerian institutions during the COVID‐19 pandemic who took a computer‐based test (CBT) instead of the conventional paper‐based examination. Discovered that Chinese nursing students were experiencing a lot of distress and irritability because of difficulties in handling technology following the learning modifications during COVID‐19. According to Cifuentes‐Faura et al. (2021), some Nigerian students' experiences during the pandemic bother around social isolation, personal well‐being and adaptation to learning outside the classroom walls.

The preceding necessitates a rapid response from the various nursing faculties and stakeholders to meet the needs of postgraduate nursing students. Goh et al. (2021) identified research/clinical skills development as one of the essential needs of nursing students during the COVID‐19 pandemic, which requires HEIs to provide comprehensive and specific support to students. Jiang et al. (2021) discovered that students' satisfaction with online learning was significantly influenced by prior experience, acceptance of learning methods, computer self‐efficacy and academic self‐efficacy. These factors are worth considering when planning support programs for students. To the best of our knowledge, there is a paucity of studies exploring the experiences of postgraduate nursing students within the Nigerian context. Hence, this study intends to explore these questions:

1. What are the educational needs of postgraduate nursing students in Nigeria?
2. What are the experiences of postgraduate nursing students in Nigeria during the COVID‐19 pandemic?

## 2 MATERIALS AND METHODS

The study employed a descriptive qualitative phenomenological approach to explore the experiences of Nigerian postgraduate nursing students and their needs during the COVID‐19 pandemic and to identify ways that can be used to improve the outcomes of their training (Moorley & Cathala, 2019). The study population comprised postgraduate nursing students from various higher educational institutions in Nigeria. Research Ethics Committee approval was obtained from the research and ethical committee of Lagos University Teaching Hospital. A purposive sampling technique was employed to select postgraduate nursing students in Nigeria whose studies were impacted by the COVID‐19 pandemic (Shorten & Moorley, 2014). This included those who experienced disruptions in their academic schedules, challenges with online learning, or other pandemic‐related obstacles. This approach ensured the inclusion of nurses from diverse backgrounds, marital statuses, types of programme, universities/colleges and areas of specialization. This approach allowed us to capture a wide range of experiences and perspectives related to the impact of the pandemic on their education. One‐on‐one semi‐structured in‐person and telephone interviews were conducted among 22 participants until data saturation was reached. The participants were from nine universities. Among these, six were federal universities, two were state universities, and one was a private university.

The inclusion criteria encompassed Nigerian postgraduate nursing students who were enrolled in any university or college in Nigeria during the COVID‐19 pandemic and who consented to participate in the study. Exclusion criteria were non‐Nigerian postgraduate nursing students and those who were not enrolled in a postgraduate nursing program during the COVID‐19 pandemic.

Data collection took place between February and April 2022. Eligible participants were approached and invited to join the study. Rapport was established between the interviewer and each participant. One of the ways rapport was established was by starting from the simple questions to the hard questions. A research information sheet containing the study background, the purpose of the study, procedure, risks and benefits, and ethical considerations was offered to each participant, after which they were required to sign a written consent form.

Each session was conducted by research assistants in a quiet room either within the department of nursing or through phone calls. The research assistants introduced themselves, stated the purpose of the interview and introduced the ground rules. The research assistants utilized a semi‐structured interview guide for data collection. The interview guide included four key sections. The introduction section explained the study's purpose, assurance of confidentiality and obtaining verbal consent. The demographic information section focused on the collection of basic demographic details such as age, gender, university type and current year of study. The interview questions focused on pre‐pandemic challenges, pandemic‐related needs, impact of COVID‐19, challenges during the pandemic, adjustments to the new normal and future innovations. The closing questions provided participants with an opportunity to add any final thoughts and reflections.

Each interview lasted around 30–45 min. All the discussions were audio‐recorded and later transcribed verbatim for data analysis. To ensure the quality of data obtained for this study, the researchers listened to every recording after each one‐to‐one interview. The researchers kept reflective journals to check biases as the research progressed. The researchers also documented new themes emerging from each interview, which enabled them to probe such in subsequent discussions (Table 2). All audio‐recorded interview files were sent to the principal investigator immediately after each session. Verbatim transcription of the discussion was done while listening to the recording.

The data were analysed using Colaizzi's Method of Qualitative Data Analysis, a systematic approach comprising seven rigorous steps aimed at deeply understanding participants' experiences from their original descriptions. To start with, the transcripts were read multiple times to achieve immersion in the data, allowing for a thorough grasp of the context and nuances of participants' narratives. Initial impressions and significant points were noted during this immersion phase. Second, significant statements were extracted from the transcripts. These statements directly related to the phenomenon under study and were identified by carefully reviewing each line of the transcript and highlighting phrases that conveyed meaningful insights. Thirdly, meanings were ascribed to these significant statements through a reflective process. Overall, we had 120 meaning units. Each statement's context was carefully considered and interpretations were made to uncover implicit meanings and the essence of what participants were conveying. These meaning units were then systematically analysed and categorized, resulting in 75 distinct codes. Next, the codes were organized into clusters of themes. Similar or related meanings were grouped together to form broader themes that emerged from the data. This thematic clustering process was validated by revisiting the transcripts to ensure that the themes accurately represented participants' experiences. Any discrepancies or ambiguities were discussed with peers to reach a consensus. The next step entailed a detailed description of the phenomenon under study. This description aimed to encapsulate the essence of participants' experiences by integrating the clustered themes into coherent narratives. Next, the structure of the phenomenon was established by refining the detailed description to highlight its fundamental elements and core aspects. This involved distilling the detailed narratives to focus on the essential themes and their interrelationships, providing a clear and concise representation of the phenomenon. Lastly, the exhaustive description was validated through feedback from participants. They were presented with the synthesized themes and descriptions and asked to review them for accuracy and resonance with their experiences. Their feedback was incorporated to refine and validate the findings, ensuring that the final description authentically reflected their perspectives (Wirihana et al., 2018). The NVIVO software was used for data analysis.

## 3 RESULTS

The participants were majorly between ages 31–50 (90.9%), mostly married (68.20%), from the three major ethnic groups in Nigeria, Yoruba, Igbo and Hausa, majorly Christians (86.40%), from four geopolitical zones in Nigeria, majorly undergoing master's degree education (72.7%), in Federal institutions (77.3%) and majorly working in educational facilities (72.7%) See Table 1.

**TABLE 1:** Socio‐demographic details of the participants.

Variables	Categories	Frequency (*n* = 22)	Percent (%)
Age	31–40	11	50.0
	41–50	9	40.9
	51–60	2	9.1
	Mean ± SD	1.59 ± 0.67	
Marital Status	Single	7	31.8
	Married	15	68.2
Ethnic Group	Yoruba	7	31.8
	Igbo	8	36.4
	Hausa	7	31.8
Religion	Christianity	19	86.4
	Islam	3	13.6
Geopolitical Zone	South‐East	7	31.8
	North Central	3	13.6
	North West	4	18.2
	South South	3	13.6
	South West	5	22.8
Educational Program	Post Graduate Diploma	1	4.5
	Masters	16	72.7
	PhD	5	22.7
Educational Facility	Federal	17	77.3
	State	3	13.6
	Private	2	9.1
Place of Work	Clinical Facility	3	13.6
	Educational Facility	16	72.7
	State Ministry of Health	3	13.6

### 3.1 Themes and subthemes

Three main themes emerged from the data analysis. The themes are challenges of Nigerian postgraduate nursing students before the pandemic, the impact of the pandemic on postgraduate nursing education in Nigeria and innovations to improve postgraduate nursing education in Nigeria. These were further elaborated under 14 sub‐themes (Table 2).

**TABLE 2:** Themes and Sub‐themes.

Themes	Sub‐themes
Challenges of Nigerian postgraduate nursing students	Paucity of funds Inadequate power supply Poor internet access Lack of infrastructure Shortage of human resources Poor mentorship of postgraduate nursing students
Impact of the pandemic on postgraduate nursing education in Nigeria	Abrupt disruption of academic programme Prolonged academic calendar Communication gap between students and their research supervisors
Innovations to improve postgraduate nursing education in Nigeria	Adoption of elearning in postgraduate nursing education Postgraduate nursing education should be well‐structured Post basic programmes should be upgraded to postgraduate programmes More universities should commence post graduate nursing programmes Provision of financial aid for students

### 3.2 Theme 1: Challenges of Nigerian postgraduate nursing students

Before the onset of the pandemic, Nigerian postgraduate nursing students faced significant challenges that impacted their educational experience and professional development. These challenges encompassed various aspects of infrastructure, support systems and resource availability, which collectively influenced the students' ability to thrive in their academic pursuits and clinical training. Seven categories of the participants' challenges regarding their needs and experiences during their postgraduate programme came to the fore. They are further detailed under the following sub‐themes:

#### 3.2.1 Paucity of funds

This subtheme underscores the significant financial challenges faced by Nigerian postgraduate nursing students before the pandemic. This challenge encompasses the affordability of school fees, study materials and access to essential resources necessary for academic and research endeavours. The quotes provided by participants highlighted the financial strain experienced by students, impacting their ability to fully engage in educational opportunities and conduct research effectively.

There's a lot we should investigate, such as funding. Funding is the major thing. Regarding funds, school fees and study materials are not very affordable for most students. I have been in the school environment for a little while now; you see, some of us still complain about the high cost of fees. (P1,40 years, PhD student)

Because of lack of funds, students cannot readily access resources that will facilitate our research. (P20, 41 years, PhD student)

#### 3.2.2 Inadequate power supply

This subtheme addresses the challenges faced by the participants due to unreliable electricity supply. This challenge impacted their ability to engage in online learning, access digital resources and complete academic tasks that require electricity‐dependent equipment. Participants highlighted the frequent disruptions caused by power outages, affecting both study sessions and the use of essential electronic devices.

You know this is Nigeria; the power supply is always a problem. Sometimes you might want to do something, but you discover there is no light. (P16, 42 years, Master's student)

One of the challenges I face is power outage. I could be on a session, and my computer goes off due to power failure. (P2, 41 years, PhD student)

#### 3.2.3 Poor internet access

This subtheme highlights the challenges faced by the participants due to inadequate or unreliable internet connectivity. This challenge significantly impacted their ability to participate in virtual classes, complete online exams and access digital learning resources essential for their studies. Participants described the frustrations and disruptions caused by network connection issues, which had adverse effects on academic performance and engagement.

There were network connection problems, especially during exams, and if that happens, you may repeat the exam; this affected my grades. By the time I log in, the exam might have started 30 minutes earlier. It can even trip off even when you are writing, and nobody waits for you; you are on your own. (P3, 50 years, Master's Student)

I had issues with poor internet connection during virtual classes. So, what I did most of the time was to move about six yards away from where I live, where they have an MTN mast; there the internet was much better. So, every 6 p.m., I had to drive to that area and park most of the time. I'll be there for four good hours to ensure I catch up with classes without disruption from the internet. (P1, 40 Years, Master's Student)

#### 3.2.4 Lack of infrastructure

This subtheme highlights the inadequacies in facilities and resources within Nigerian institutions offering postgraduate nursing programs. Participants frequently noted deficiencies in physical infrastructure, such as laboratories and libraries, which are critical for practical training, research and accessing scholarly resources. These shortcomings significantly impacted the educational experience and academic opportunities available to postgraduate nursing students.

Postgraduate nursing programmes started not long ago; some schools are patching structures not well equipped for the Department of Nursing. Also, the issue of laboratory for certain practical and other higher research is lacking in most of our postgraduate programmes. (P10, 49 Years, Master's Student)

Another thing I can think about is the lack of access to a quality library. Several journals are not yet subscribed to by the University, which is one of the challenging issues that postgraduate nursing students face. (P9, 32 Years, Master's Student)

#### 3.2.5 Shortage of human resources

This subtheme presents the challenges faced by the participants due to insufficient qualified faculty and clinical preceptors. This shortage affects the quality of education and mentorship available to students pursuing advanced nursing degrees. Participants expressed concerns about the lack of experienced PhD holders and competent tutors within the nursing field, which impacts the delivery of education and practical training.

…also from my little experience as a PhD student, our lecturers are not vastly experienced in carrying on with this programme because everything is still like trial and error. We also want to have PhD holders. (P12, 34 Years, PhD student)

Another problem was the lack of competent tutors in the field of study. Sometimes, some lecturers who teach nurses are not nurses, so they sometimes want to apply things from their discipline to the nursing profession. This hinders clear understanding of what is expected of you in the delivery of care to patients. (P19, 40 Years, Master's Student)

Manpower is an issue. Now that the Nursing and Midwifery Council of Nigeria wants to move most of the post‐basic programmes to the university setting where they will now be postgraduate programmes. The concern now is who would be the lecturers?' We are short of the workforce; we do not have many professors in Nursing. Unlike other programmes, we have few universities running nursing programmes at a postgraduate level as a result of lack of human resources. (P1, 40 Years, Master's Student)

The number of lecturers is poor. We need more hands. I am sorry to say this because of the Nigerian factor or what; everybody is running out of the country in search of greener pastures, of which, if I see my way today, I will leave too. So they should do the necessary things so the nurses here can stay back. (P20, 41 Years, PhD Student)

#### 3.2.6 Poor mentorship of postgraduate nursing students

This subtheme highlights the inadequate support and guidance provided to students by their academic supervisors or mentors. Participants expressed concerns about the lack of effective mentorship relationships, citing issues such as supervisors being overloaded with responsibilities, which impacted their availability and commitment to support students' academic and research endeavours.

Another challenge postgraduate nursing students face is the lack of good mentorship and supervisor relationships. I have discovered that most of our supervisors are overloaded with work, affecting their commitment to their supervisee's work. (P9, 32 Years, Master's Student)

Postgraduate nursing students lack academic mentors during their programme, which can be quite challenging… (P12, 34 Years, PhD student)

### 3.3 Theme 2: Impact of the COVID‐19 pandemic on postgraduate nursing education in Nigeria

This theme explores the significant disruptions and challenges faced by postgraduate nursing education in Nigeria because of the COVID‐19 pandemic. It focuses on how the pandemic abruptly interrupted academic programs, extended academic calendars and exacerbated communication gaps between students and their research supervisors. The sub‐themes highlight the profound impact of the pandemic on the continuity of education, academic planning and mentorship within postgraduate nursing programs. The subthemes are:

#### 3.3.1 Abrupt disruption of the academic programme

This subtheme highlights the sudden and unprepared halt in educational activities within Nigerian postgraduate nursing programs due to the COVID‐19 pandemic. Participants described the lack of institutional readiness and technical preparedness, resulting in a significant loss of academic progress and opportunities.

…in fact, there was no arrangement officially to be able to reach out to students. There were no proper arrangements as we saw in most private universities that had already adopted e‐learning. Mine is a federal university; there was no arrangement for us to continue running the programme. (P10, 49 Years, Master's Student)

By the time we resumed, so many had lost it all. It was a wasted session. Some lecturers were not technically balanced to disseminate information to the students. Some students, on the other end, were not technically inclined to. So, a whole session was lost due to the COVID‐19 pandemic. (P19, 40 Years, Master's Student)

We would have graduated and maybe gone for further education; maybe we would have been doing our PhD now. As I told you, I am retiring this year, and I should have been done with my master's and gone for my PhD, but now I am still struggling with my master's kai…. (P6, 59 Years, Master's Student)

#### 3.3.2 Prolonged academic calendar

This subtheme explores the prolonged timelines of postgraduate nursing programs in Nigeria, exacerbated by disruptions caused by the COVID‐19 pandemic. Participants expressed frustration and uncertainty regarding their graduation timelines, attributing delays to lockdown measures, halted academic activities and challenges in completing coursework and research requirements.

I discovered that some were admitted before we could not graduate on time, but their contemporaries in other departments were through with their programmes and re‐admitted for PhD. Nursing departments all over the country should wake up so that their students can graduate on time. (P10,49 Years, Master's Student)

The first thing that changed was the duration of the programme. Right now, I do not even know when I will be graduating. Even though I am done with my coursework, I still don't know when I will graduate. (P11, 30 Years, Master's student)

I commenced my programme during the 2018/2019 academic session, and I was supposed to round off by 2020, but up till now, we have not even finished because of the COVID lockdown. The pandemic affected us, it delayed everything, and nobody went to school. We are still struggling to graduate. (P6, 59 Years, Master's Student)

The master's programme is supposed to last at least a year and six months, but it is now getting to three years, and the person cannot go on with the research, and sometimes you will see sometimes it takes up to five years to successfully round off just ordinary master's programme. (P20, 41 Years, PhD Student)

#### 3.3.3 Communication gap between students and their research supervisors

This subtheme presents the challenges faced by postgraduate nursing students in Nigeria regarding inadequate access and interaction with their supervisors. Participants expressed frustrations over delayed feedback, limited virtual communication effectiveness and the absence of in‐person guidance, which hindered their research progress and academic support.

My supervisor was not accessible at some point, so things we had to learn physically could not be learnt. Sending my work for correction and returning it to my supervisor was very challenging. Unlike when you go to a physical office to meet your supervisor to do what was necessary. (P14, 32 Years, PGDE)

We had to always plead with our supervisors to read our research proposal during the pandemic to enable us to continue our work. Some will hold on to your write‐up for six months without feedback. I can say that during the pandemic, the virtual stuff did not work for my research supervision. (P15, 34 Years, Master's Student)

Well, my need presently is to have access to my supervisor. It might sound somehow, but my need presently is to have better access to my supervisor because COVID‐19 prevented physical or one‐on‐one interaction, and this affected me in a way. (P22, 35 Years, PhD Student)

The pandemic did not give my supervisor enough time to check my work. Sometimes, this may mean that you are on your own. You know, meeting a supervisor physically is different from communicating online; if you meet the supervisor physically, it will give room for them to teach you, but there is usually no time to learn online. (P3, 50 Years, Master's Student)

### 3.4 Theme 3: Innovations to improve postgraduate nursing education in Nigeria

This theme focuses on innovative approaches aimed at enhancing postgraduate nursing education in Nigeria. It highlights initiatives and strategies aimed at addressing existing challenges and improving the quality, accessibility and sustainability of education for postgraduate nursing students. They are further explored through the following sub‐themes:

#### 3.4.1 Adoption of e‐learning in postgraduate education

The subtheme explores the perspectives of participants regarding the integration of online learning methods into postgraduate nursing education in Nigeria. Participants emphasized that they wanted e‐learning to be incorporated into their academic programs due to its numerous benefits such as providing flexibility, resilience during emergencies like pandemics and enhancing educational accessibility and effectiveness.

If there is anything the school or university should do, it should be to make lectures a mixture of physical contact and online lectures, that is, the adoption of e‐learning. It could help a lot, so if there should be any outbreak of any pandemic in the future, physical lectures can just be swapped to online lectures, online assessments and all that without disrupting the school calendar. (P11,30 Years, Master's Student)

We need to improve our lecture methods, and we need more modernized ways of conducting online lectures, which other schools of learning have adopted and my school has not. We need ICT personnel to coordinate those things; we need well‐trained lecturers who are up to standard and trained to handle ICT; not everyone is competent to handle online stuff. (P18,40 Years, Masters Student)

#### 3.4.2 Proper structuring of postgraduate nursing education

This subtheme addressed the perspectives of participants on the structural framework of postgraduate nursing education in Nigeria. It highlights the current challenges related to program structure and advocates for improvements that align with international standards and enhance career progression and leadership roles for nurses.

I want the postgraduate nursing programme to be like clinical residency. There should be courses based on clinical programmes so we can start having consultants in nursing the way we have medical residency. I hope that one day, nursing will also have this programme. So, we will begin to have consultant nurses. (P11, 30 Years, Master's Student)

Postgraduate nursing education should be structured in a way that PhD holders will be absorbed into managerial ranks in nursing. People like that can be part of the decision‐makers in the hospital. In my workplace, supervisors usually meet regularly with nursing leaders, so people like that can be called occasionally into the discussion desk to give their views about patients and outcomes of care. (P13, 35 Years, Master's Student)

#### 3.4.3 Upgrading post‐basic to postgraduate nursing programmes

This subtheme explores participants' perspectives on the transformation of post‐basic nursing programmes into postgraduate programmes in Nigeria. Participants advocated for elevating the educational standards of these programmes to align with international norms and enhance the professional qualifications and capabilities of nurses.

I wish to see all those post‐basic programmes being offered at postgraduate level instead. (P2,32 Years, Master's Student)

… we have about 17 post‐basic nursing programmes in Nigeria; I want to see them turned into master's programmes. For example, masters in ICU NURSING and so on. (P7, 39 Years, Master's Student)

#### 3.4.4 Commencement of postgraduate nursing programmes in more universities

This subtheme examines participants' views on expanding the availability of postgraduate nursing programmes across universities in Nigeria. Participants expressed frustration over limited admission opportunities and advocated for the establishment of more programmes to meet the growing demand for advanced nursing education.

Every university should start postgraduate nursing programmes because the demand is high. I remember it took me many years to gain admission; I went to a federal university in the West and aced their exams but could not be admitted because there were few slots for northerners. I wish more universities could start postgraduate nursing programmes. (P10, 49 Years, Master's Student)

They should give support, and they should make sure the courses are being accredited, so that if you have various institutions doing nursing, at least the choice will not be limited. (P17, 40 Years, Master's Student)

Every university, private and government institutions, the NUC should help them start postgraduate courses in nursing. Most universities have not started offering postgraduate programmes. (P20, 41 Years, PhD Student)

We have so many people who desire to have a postgraduate education in Nursing but are limited, even in the number of schools that offer postgraduate programmes in Nigeria. I can recount about ten times I should have been admitted to federal universities in the West and North. I discovered that even after getting admission in terms of resources, there are not enough resources. (P7, 39 Years, Master's Student)

The major challenge is even gaining admission in Nursing because there are not many institutions running master's and PhD programmes, so securing admission is a challenge. (P8, 40 years, Master's student)

#### 3.4.5 Provision of financial aid for students

This subtheme explores participants' perspectives on the critical need for increased financial support for postgraduate nursing students in Nigeria. Participants highlighted the challenges of funding their education and emphasized the importance of scholarships, grants, and other forms of financial aid to facilitate their academic pursuits.

Scholarship opportunities should be made available for willing postgraduate nursing students who want to embark on postgraduate programme. (P17, 40 Years, Master's Student)

Postgraduate students should be given access to grants. It is assumed that most postgraduate programmes are research‐based. The government can help give students grants to enable them to fund their research. (P5, 41 Years, PhD)

The first thing is financial assistance. I think financial assistance should be made available for students. The country is bad, and the economy is bad. They should assist students in financing their education. Even if they cannot pay, let there be student loans…. (P15, 34 years, Master's student)

## 4 DISCUSSION

This study explored the needs and experiences of postgraduate nursing students in Nigeria during the COVID‐19 Pandemic. Twenty‐two postgraduate nursing students selected using the purposive sampling technique were interviewed. Three themes and 14 sub‐themes emerged from the analysis. Various challenges experienced by postgraduate nursing students in Nigeria came to the fore. Findings suggest that most of them verbalize funding as a significant need. This is not surprising given the economic realities in a developing country like Nigeria, which is one of the nations with the highest number of people living in extreme poverty (Esan et al., 2022). Similarly, a study by Havenga and Sengane (2018) reinforced that paying tuition fees, data purchases, and other expenses was financially demanding. In trying to meet the program's financial demands, many postgraduates have jobs and businesses but cannot run them smoothly because of the burden of physical lectures.

Not until the pandemic did anecdotal evidence reveal that teaching and learning activities in Nigerian Universities were limited to face‐to‐face classes and transmitting to online classes came with challenges. In the findings of Mpungose (2020), conducted among South African Students, students did not like the idea of an online class because they lacked the resources, such as good internet, power supply, etc, to connect to e‐classes. Although internet usage increased in Nigeria during the COVID‐19 pandemic (Akingbade et al., 2023), the lack of power supply and the cost of the internet are still crucial challenges faced by Nigerians (Akingbade et al., 2022; Ogbeide et al., 2022). Twenty‐four postgraduate students in another study agreed that the infrastructure was inadequate (Amina & Lucky, 2016). Shortage of lecturers and lack of competent tutors are some of the challenges experienced by postgraduate students. Although baccalaureate nursing education began in Nigeria at the Faculty of Nursing, University of Ibadan, in 1965 (Arowolo et al., 2023), evidence suggests that there is still a shortage of lecturers in citadels of nursing in Nigeria, which has also been adversely affected by the massive brain drain (Adejumo et al., 2023). This is also the case in Iran, where they face a smaller ratio of lecturers compared to students in the department (Hajihosseini et al., 2017). Mentorship was another need of postgraduate nursing students. Everyone at some point in their lives needs someone who can hold their hand through the journey; meanwhile, there are still concerns about the poor state of mentorship in nursing in Nigeria. Adejumo et al. (2023) reported that young Nigerian nurses lamented the poor state of mentorship in Nigerian nursing, and they verbalized the need for more support through their career trajectories. The importance of mentorship in postgraduate nursing has been documented. Moghaddam et al. (2019) demonstrated that the mentorship program enhanced their knowledge in research, which in turn fast‐tracked their thesis writing and graduation from school.

Considering the impact of the COVID‐19 pandemic on postgraduate nursing education, the sudden disruption of the academic program did not come easy for most participants in this study as some of the institutions in Nigeria were unprepared and did not know how best to continue the program despite the happenings. Despite the sudden disruption, postgraduate students in Saudi Arabia were pleased with the new way of learning; it came easy for them as it allowed them to continue their program without delay (Hemdi, 2021), unlike in Bangladesh, where students were displeased with the sudden decision to shut down schools which concurred with the findings in this study (Dutta & Smita, 2020). As a developing country, Nigeria found it challenging to adjust to the new normal, extending the proposed planned graduation date. A study conducted in a public university in Nigeria by Aiyedun and Ogunde (2020) agreed that the pandemic affected the academic calendar. Furthermore, considering the shutting down of schools, students and their research supervisors could not communicate as they used to before the pandemic. In this study, postgraduate students lamented how reaching out to their supervisors became challenging. In their study, discovered that the challenges due to the long‐distance type of supervision following the pandemic were real and experienced by even research students in Pakistan.

The third theme of this study concentrated on innovations to improve postgraduate nursing education in Nigeria. Most respondents in this study suggest that postgraduate nursing programs should be more open to adopting e‐learning. This is important, as a recent study revealed that nurses perceived an online nursing research conference held in Nigeria during the COVID‐19 pandemic as highly impactful and satisfactory (Adesuyi et al., 2023). However, the need for training in the use of computers was noted. Agreeing with this is the study of Dhawan (2020), who found out that mixing physical and remote learning encourages the students' learning skills. Proper structuring of postgraduate nursing education, upgrading post‐basic programs to postgraduate programs, accrediting more Universities to run postgraduate programs and approving more funds for postgraduate students in Nigeria are innovative ways suggested by respondents to move postgraduate nursing education forward.

### 4.1 Strength and limitation

To the best of our knowledge, this study is the first in Nigeria to explore the experiences of postgraduate nursing students across various geopolitical zones during the COVID‐19 pandemic. However, due to the small sample size, this might not reflect the complete picture. Nevertheless, policymakers and stakeholders in the Nigerian educational system will find these findings helpful and valuable.

## 5 CONCLUSION

This study has highlighted the experiences of postgraduate nursing students in Nigeria during the COVID‐19 pandemic. It is obvious that post‐graduate nursing programs in Nigeria still need reforms. Collective efforts are essential to overcome the challenges identified in this study and enhance the learning experience for postgraduate nursing students in Nigeria.

## AUTHOR CONTRIBUTIONS

**Oluwadamilare Akingbade:** Conceptualization; methodology; writing – review and editing; formal analysis; data curation; investigation; writing – original draft; supervision. **Victoria O. Faremi:** Project administration; conceptualization; investigation; writing – original draft; methodology. **Chioma J. Eze:** Methodology; writing – review and editing. **Chioma B. Eze:** Conceptualization; writing – original draft; data curation. **Esther Oluwasola:** Investigation; conceptualization; writing – original draft. **Samuel A. Olawoore:** Conceptualization; investigation; writing – original draft; data curation. **Victoria Adediran:** Writing – original draft; conceptualization; investigation; validation. **Oluwatobi B. Kolawole:** Writing – original draft; conceptualization; data curation. **Emmanuel O. Adesuyi:** Supervision; conceptualization; methodology; writing – original draft; writing – review and editing; investigation; validation.

## ACKNOWLEDGEMENTS

We appreciate all respondents who took time to participate in the study. We also appreciate the Institute of Nursing Research, Osogbo, Osun State, Nigeria, for the technical support while the project lasted.

## FUNDING INFORMATION

The authors received no funding for this study.

## CONFLICT OF INTEREST STATEMENT

The authors declare no conflict of interest.

## ETHICS STATEMENT

Ethics approval was sought and obtained for the study from research and ethics committee of Lagos State University Teaching Hospital, Nigeria. The participants were given detailed information about the study, and they completed a consent form afterward. They were assured that participation is voluntary. Similarly, they were assured of anonymity and confidentiality.

## Data Availability

Manuscript data are available upon reasonable request to the corresponding author.
